# Characterization of a cohort of metastatic lung cancer patients harboring *KRAS* mutations treated with immunotherapy: differences according to *KRAS G12C* vs. *non-G12C*

**DOI:** 10.3389/fonc.2023.1239000

**Published:** 2023-10-17

**Authors:** Lucía Notario, Marc Cucurull, Gabriela Cerdà, Carolina Sanz, Enric Carcereny, Ana Muñoz-Mármol, Ainhoa Hernández, Marta Domènech, Teresa Morán, Montse Sánchez-Céspedes, Marta Costa, Jose-Luis Mate, Anna Esteve, Maria Saigí

**Affiliations:** ^1^ Medical Oncology Department, Catalan Institute of Oncology (ICO)-Badalona, Germans Trias i Pujol University Hospital (HUGTiP), Badalona-Applied Research Group in Oncology (B-ARGO), Germans Trias i Pujol Research Institute (IGTP), Badalona, Barcelona, Spain; ^2^ Pathology Department, Germans Trias i Pujol University Hospital (HUGTiP), Germans Trias i Pujol Research Institute (IGTP), Badalona, Barcelona, Spain; ^3^ Cancer Genetics Group, Josep Carreras Leukaemia Research Institute (IJC), Badalona, Barcelona, Spain; ^4^ Badalona-Applied Research Group in Oncology (B-ARGO), Germans Trias i Pujol Research Institute (IGTP), Badalona, Barcelona, Spain; ^5^ Statistics Department, Catalan Institute of Oncology (ICO)-Badalona, Applied Research Group in Oncology (B-ARGO), Germans Trias i Pujol Research Institute (IGTP), Badalona, Barcelona, Spain

**Keywords:** non-small cell lung cancer, lung adenocarcinoma, KRAS, PD-L1, immunotherapy

## Abstract

Approximately 20% of lung adenocarcinomas harbor activating mutations at *KRAS*, an oncogene with the ability to alter the tumor immune microenvironment. In this retrospective study, we examined 103 patients with *KRAS*-mutant lung adenocarcinoma who were treated with immunotherapy-based regimens and we evaluated the clinical outcomes according to PD-L1 expression and the type of *KRAS* mutation. Among all patients included, 47% carried *KRAS G12C* mutation whereas 53% harbored *KRAS* non-G12C mutations. PD-L1 status was available for 77% of cases, with higher expression among KRAS G12C tumors (p = 0.01). Better overall survival and progression-free survival were observed in high PD-L1 expression tumors, regardless of *KRAS* mutation type. The heterogeneous nature of KRAS-mutant tumors and the presence of other co-mutations may contribute to different outcomes to immunotherapy-based strategies.

## Highlights

- *KRAS G12C* mut LuADs are significantly associated with high PD-L1 expression.
- Better clinical outcomes are associated with high PD-L1 expression, regardless of *KRAS* mut type.
- A subset of long-term responders (LTR) to IT-based regimens were enriched with *KRAS G12C* mut and high PD-L1 expression.

## Introduction

Lung adenocarcinoma (LuAD) harbors a significant number of targetable oncogenic mutations among lung cancer. The most common oncogenic mutations are found in *KRAS*, occurring in 20%–25% of the cases. These mutations primarily affect codons 12 (85%) and 13 (10%), found in exon 2, and codon 61 (5%), found in exon 3. The *KRAS G12C* mutation, resulting in a change from glycine to cysteine, prevails in 43% of the cases and is associated with tobacco exposure. In contrast, non-smokers, commonly exhibit G12D mutations, a change from glycine to aspartic acid, and G12V mutations, a change from glycine to valine (1).

Currently, the standard first-line treatment for patients with *KRAS G12C* LuADs involves IT-based regimens, either combined or not with platinum-based chemotherapy (ChT) according to PD-L1 expression levels. Mazieres et al., in a retrospective cohort, demonstrated that *KRAS*-driven tumors express higher rates of PD-L1 and present higher tumor mutational burden compared with other oncogenic alterations, suggesting that it might predict better responses to IT (2). However, the study did not evaluate the existing differences based on the type of *KRAS* mutation. On the other hand, most phase III pivotal trials with IT did not stratify by *KRAS* status, and the efficacy of IT according to *KRAS* mutation subtype remains to be determined (3).

Recently, the emergence of novel allosteric inhibitors of KRAS G12C is expected to change the paradigm of treatment approach for these tumors. Phase I/II trials with sotorasib and adagrasib presented an overall response rate (ORR) of 32% and 45%, respectively, along with an acceptable toxicity. As a result, these inhibitors have received the approval of the Food and Drug Administration (FDA) for the treatment of patients with *KRAS G12C* mutations after progression to initial therapy (4, 5). To further enhance the outcomes, novel drugs such as BI-3406 which disrupts the interaction of SOS1-KRAS, as well as TNO155, which inhibits SHP2, a protein that integrates growth and differentiation signals from receptor tyrosine kinases into the RAS/MAPK cascade, are being evaluated. These drugs in combination with sotorasib or adagrasib are being studied to improve treatment outcomes (5). Additionally, there have been promising findings from preclinical models combining PD-1 inhibitors with KRAS G12C-specific inhibitors. These combinations are being addressed in clinical trials (NCT04613596).

As a result, targeting KRAS beyond KRAS G12C inhibitors has emerged as a significant and rapidly evolving area of research. This refers not only to the development of novel therapeutic strategies targeting *KRAS* but also to the immunoregulatory role of *KRAS* and the effectiveness of current immunotherapies in *KRAS-*driven tumors, which has not been directly addressed in the literature. Of note, *KRAS G12C*-mutant tumors are commonly associated with tobacco exposure and exhibit higher tumor mutational burden, which might predict better responses to IT (6). We hypothesized that those patients might present better clinical outcomes to IT-based therapies.

In this work, we aim to study the clinical outcomes of existing immunotherapies based on the type of *KRAS* mutation and PD-L1 expression levels. We will examine a cohort of patients with metastatic *KRAS*-mutant tumors treated with IT-based regimens in our daily practice.

## Methods

### Study population

A medical record search was used to identify patients treated at the Catalan Institute of Oncology (ICO)-Badalona with a primary tumor diagnosis of NSCLC harboring *KRAS* mut and treated with IT-based regimens for metastatic disease, from June 2013 to June 2020. Clinical data were retrospectively collected, and patient consent forms were obtained with the approval of the local Institutional Review Board (PI-19-275).

### Molecular analysis and PD-L1 expression

The *KRAS* mutation status was determined by analyzing the primary tumor. The tumor tissue samples were tested by KRAS Idylla Mutation Test (Biocartis), a real-time PCR test designed for the identification of mutations in codons 12, 13, and 61; in the most recent cases (2020–2021), they were tested by the NGS panels: Oncomine Solid Tumour, Oncomine Focus Assay, or Oncomine Comprehensive Assay (Thermo Fisher) which includes 22, 52, and 164 genes, respectively, involved in lung cancer pathogenesis. The PD-L1 status in tumor cells was determined by immunohistochemistry (IHC) assay (Ventana clone SP263), and it was categorized as follows: negative <1%, low 1%–49%, and high 50%–100% expression.

### Statistical analysis

Clinical characteristics, *KRAS* mutation type, and line of IT treatment were collected for all patients. We classified patients into two groups based on the *KRAS* mutation type: G12C or non-G12C. Baseline characteristics were compared using the chi-square and Fisher’s exact test for categorical data. Survival Kaplan–Meier model was used to estimate survival, and medians were compared between groups using the log-rank test. Progression-free survival (PFS) to IT was calculated from the time of IT initiation to date of disease’s progression or death, whichever occurred first. Overall survival (OS) was calculated from the time from starting IT treatment to date of death or last follow-up. The assessment of best overall response (ORR) to IT was performed according to RECIST 1.1 criteria, and response rates were compared between groups using the chi-square test. We defined a subset of long-term responders (LTR) to IT, defined as those patients who did not progress within 24 months after IT treatment initiation.

## Results

### Clinical and molecular characteristics according to *KRAS* mut type

We identified 103 patients with metastatic non-small cell lung cancer harboring *KRAS* mutations from June 2013 to June 2020, n = 47 *KRAS G12C*, n = 52 *KRAS non-G12C*, n = 4 unknown. PD-L1 was available in 78 cases (77%). Clinical and molecular characteristics according to *KRAS* mut type are shown in **Table 1**. The distribution of *KRAS* mutations in our non-G12C sub-cohort (n = 52) was as follows: G12V (n = 16), G12A (n = 12), G12D (n = 8), G13C (n = 5), and the frequency of the rest of mutations (G12S, G12F, G13D, Q61H) were below 5.

**Table 1:** Patient characteristics by KRAS mutation (*KRAS G12C* vs. *non-G12C*).

	KRAS G12C n = 47	KRAS non-G12C n = 52	p-value
**Median age at diagnosis**	63	61	NA
Gender			
Male Female	32 (32%) 15 (15%)	41 (41%) 11 (11%)	p = 0.224
Smoking status			
Current or former Never	46 (46%) 1 (1%)	50 (51%) 2 (2%)	p = 0.883
Performance status			
ECOG 0-1 ECOG 2-3	45 (46%) 1 (2%)	48 (91%) 5 (9%)	p = 0.142
PD-L1	*n=36*	*n=40*	
Negative Low High	6 (8%) 5 (6%) 25 (33%)	13 (17%) 13 (17%) 14 (18%)	p = 0.011
Line of treatment with IT	n = 47	n = 52	
First line Second line ≥Third line	30 (30%) 11 (11%) 6 (6%)	25 (25%) 24 (24%) 3 (3%)	p = 0.049
First-line IT, schedule of treatment	n = 30	n = 24	
IT monotherapy Combination with ChT-IT IT combination	19 (34%) 6 (11%) 5 (9%)	11 (20%) 10 (18%) 3 (5%)	NA
Percentage of retreatments with IT	3 (6%)	5 (10%)	NA

IT, immunotherapy; ChT, chemotherapy; ns, not significant; NA, not applicable.

### Clinical outcomes in *KRAS* mutant patients treated with IT

All patients included in the study were treated with IT for advanced disease: 54% in the first line, 36% in the second line, and 10% in the third or further lines. Overall, 19 patients (20%) were treated within clinical trials. Treatment schedules included different IT-based regimens at that period for the first line: ChT-IT (30%) with platinum-based doublet; IT–IT (15%); and/or IT alone (55%), the anti-PD(L)-1-based regimen being the most prevalent one (**Table 1**).

**Figure 1:**
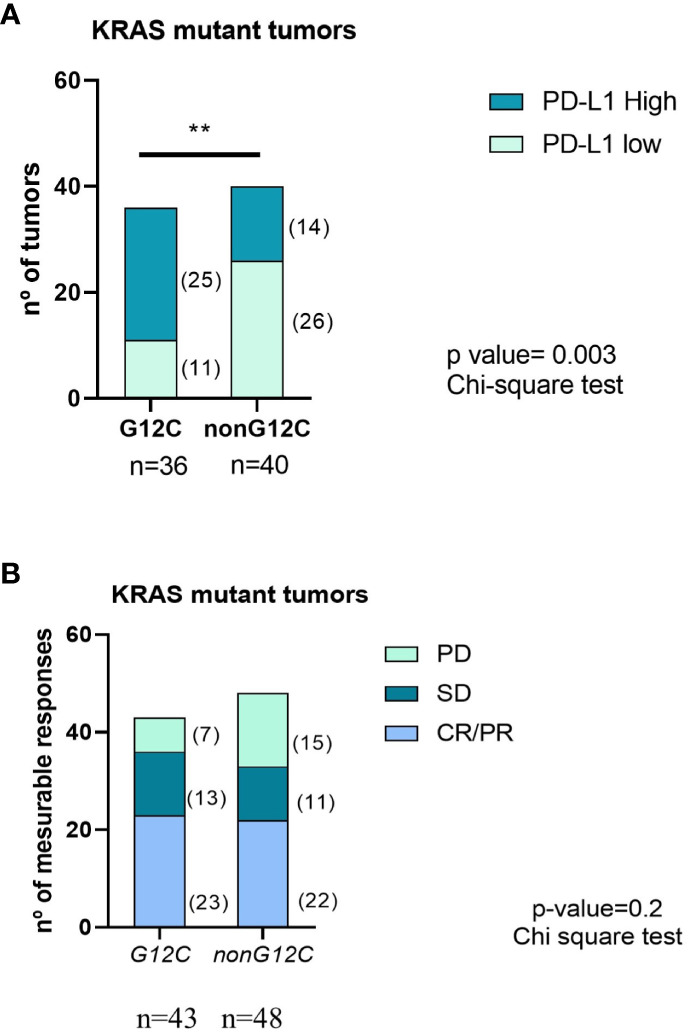
PD-L1 status **(A)** and ORR to IT **(B)** according to KRAS mutation type. PD, Progressive disease; SD, Stable disease; CR/PR, Complete/Partial response. ** means statistically significant with p-value <0.005 using chi-square test.

PD-L1 status was available in 77% of cases: 39% high, 19% low, and 19% negative. High PD-L1 (≥50%) was predominantly found in *KRAS G12C* vs. *non-G12C* (64% vs. 36%, p = 0.01). However, no statistically significant differences were observed in the overall response rate (ORR) to IT according to *KRAS* mut type: 49% of patients with KRAS G12C obtained partial or complete response compared with 42% in the non-G12C group, p value = 0.2 (**Figure 1**).

**Figure 2:**
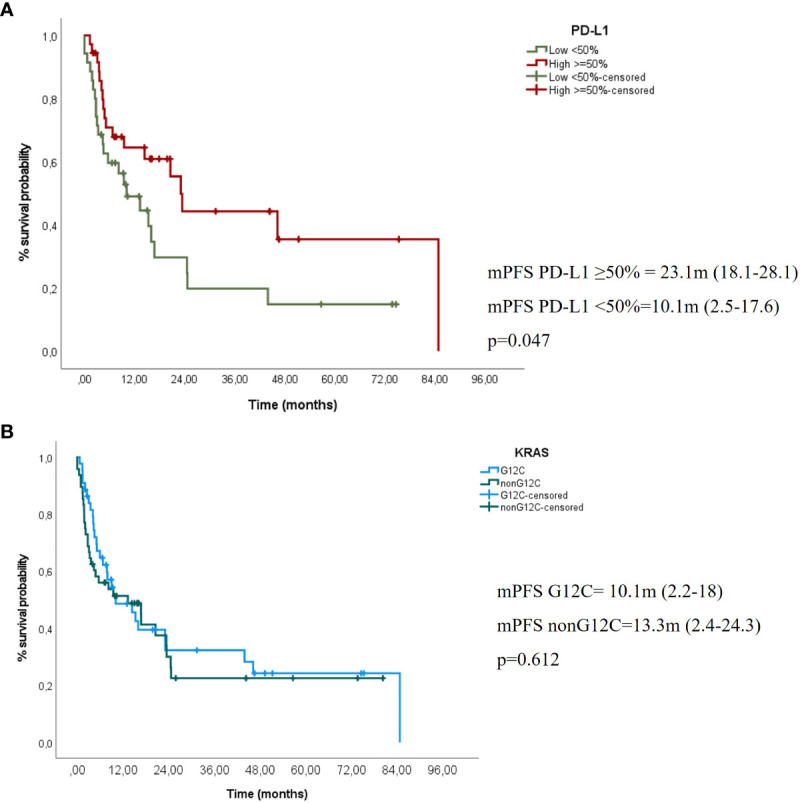
**(A)** mPFS to IT according to PD-L1 expression (high ≥50% vs. negative/low <50% **(B)** mPFS to IT according to *KRAS* mutation type (KRAS G12C vs. non-G12C).

After a median follow-up of 26.5 months (m), the mPFS of the entire cohort was 13.3 m (95% CI 5.6-20.9) and the mOS was 17.9m (95% CI 15.5–20.3). Significant differences were observed in mPFS to IT according to PD-L1 expression, regardless of the line of treatment they received the IT: 23.1 m (95% CI 18.1–28.1) in PDL1 ≥50% vs. 10.1 m (2.5–17.6) in PDL1 <50% (p-value 0.045). However, we could not demonstrate significant differences in mPFS to IT according to *KRAS G12C* vs. no-*G12C*: 10.1 m (2.2–18) vs. 13.3 m (2.4–24.3), p = 0.612, respectively (**Figure 2**). No significant differences in median overall survival (mOS) were neither observed in *G12C* vs. *non-G12C*: 17.9 m (16.6–19.2) vs. 20.6 m (12.6–28.5), p = 0.39.

In addition, in the most recent cases available for NGS (n = 30), we could determine *KRAS* mutant allele frequency, which in our cohort varies from 7.1% to 84.9%, with a mean value of 40.15%. Notably, no statistically significant differences were observed in terms of PFS or OS when employing the mean as a threshold (data details are not presented).

Finally, we identified a subset of LTR to IT (n = 17, 16%). Although not significant, they were enriched with *KRAS G12C* mutations (64%, p-value = 0.09) and high PDL1 expression (57%, p-value = 0.1) compared with the non-LTR, with no significant clinical differences.

## Discussion

In our cohort, we observed that tumors harboring *KRAS G12C* mutations were significantly associated with higher expression of PD-L1, as compared with *KRAS non-G12C.* No significant differences were observed according to the smoking habit or clinical characteristics. We also observed that patients with high PDL1 expression presented better mPFS to IT-based regimens compared with low PD-L1, regardless of the line they received the IT. However, we did not observe significant differences in mPFS to IT according to *KRAS* mutation type, despite the tendency of KRAS G12C to present better ORR to IT as compared with *KRAS non-G12C*.

Several phase III trials evaluating the efficacy of IT in NSCLC did not stratify by *KRAS* status, and only *post-hoc* analyses have been performed on that subset. Results remain controversial. While IT alone given as a first-line therapy seems to favor *KRAS*-mutant tumors compared with *KRAS-*wild type, no differences were observed when IT is given in further lines of treatment (3, 7) Another study from real-world data published by Frost et al. from a multicenter and retrospective study evaluated the efficacy of first-line pembrolizumab in 119 patients with *KRAS*-mutant LuADs with high PD-L1 expression (≥50%). Co-mutations in *TP53* were also evaluated. Patients with *KRAS G12C/TP53* had significantly higher ORR (100% vs. 27.3%; p = 0.003) and longer mPFS (33.3 vs. 2.8 months; HR, 0.18; 95% CI: 0.06–0.53; p = 0.002) than tumors with *KRAS non-G12C/TP53* mutations suggesting that *KRAS G12C* present better outcomes to immune-based therapies depending also on the co-mutation partners (8).

We also observed that the benefit of using IT was maintained for a subset of patients for at least ≥24 months after initiating IT. These patients, known as long-term responders (LTR), constitute 16% of our cohort population and were predominantly *KRAS G12C* and enriched with high PDL1 expression, although no significant differences were observed. The available literature lacks substantial information regarding tumor and patient’s characteristics of the LTR, although a few authors have suggested a potential association with adenocarcinoma histopathology and high PD-L1 expression (9).

One of the limitations of our study is the heterogeneity of the IT-based regimens that patients have received, which impairs to reach definitive conclusions. Only 16 patients received ChT-IT for the first-line setting (30%), which nowadays is the standard of care for tumors with PD-L1 <50%, regardless of the *KRAS* mutation status. Another caveat is the lack of the NGS profile for most of the patients included in the study, which was only performed in the most recent cases (2020–2021) due to diagnostic protocols in our daily clinical practice. Currently, next-generation sequencing (NGS) is the gold standard for molecular diagnosis in lung cancer since it provides a broad genetic information that helps to determine the therapeutic options. Optimizing novel panels including a wide range of genes related with carcinogenesis are becoming the standard of care. However, despite the advantages of the NGS technology, access to NGS panels varies broadly among the different areas and health systems worldwide. Co-mutations such as *STK11, KEAP1*, or *TP53* are emerging as predictive markers of response to IT, particularly in those patients with *KRAS G12C* mutations (10). In addition, in contrast to KRAS Idylla real-time PCR, NGS panels allow us to identify the KRAS mutant allele fraction, although in our cohort, additional subanalysis stratifying by *KRAS* mutant allele fraction did not allow to elucidate relevant differences in clinical outcomes. It is becoming essential to assess the genetic profile to predict different outcomes when testing different therapeutic strategies.

Another relevant topic to be addressed is the predictive value to PD-(L)1 blockade among KRAS non-G12C mutations. In this current work comprising more than 2,000 *KRAS* mutant LC patients, Ricciuti et al. show that *KRAS G12D* mutant patients harbor distinct clinical, genomic, and immunologic features and present worse clinical outcomes to PD-(L)1 blockade. Owing to the limited size of our subcohort, definitive conclusions referring to this aspect could not be reached. These inquiries continue to be of considerable interest and merit in-depth exploration within more extensive patients cohorts (11). Finally, in four cases, the *KRAS* mutation subtype was unknown because we could not access this piece of information. Those patients were remitted from other hospitals, and this constitutes another caveat of the retrospective nature of our study.

On the other hand, the strength of this study is the sample size from a multidisciplinary oncologic institution, and the long-term follow-up for all the patients included, which will help to elucidate the role of current IT in *KRAS*-mutant LuAD patients and the impact on OS, including a subset of LTR, in the era of the incorporation of KRAS G12C-specific inhibitors.

Future directions in the therapeutic landscape will focus on how to integrate IT with KRAS G12C inhibitors or panKRAS inhibitors. Recently the first data of the Codebreak 100/101 study, evaluating the combination of anti-PD(L)1 pembrolizumab or atezolizumab with sotorasib in *KRAS G12C-*mutant patients, showed promising results and represents a novel potential strategy. However, the balance between efficacy and toxicity with the combination, particularly grade 3–4 hepatotoxicity, remains crucial in this setting (NCT03600883, NCT04185883) (12).

In conclusion, despite that no significant differences were observed in IT-based regimens in lung cancer patients according to the type of *KRAS* mutation (G12C vs. non-G12C), efforts to find novel predictive biomarkers in addition to PD-L1 for *KRAS* mutant patients will help to tailor treatment in this specific population and offer them rationally designed therapeutic strategies combining both IT-based regimens with targeted therapy.

## Data availability statement

The original contributions presented in the study are included in the article/supplementary material. Further inquiries can be directed to the corresponding author.

## Ethics statement

The studies involving humans were approved by Germans Trias i Pujol Institutional Review Board (PI-19-275). The studies were conducted in accordance with the local legislation and institutional requirements. The human samples used in this study were acquired from a by- product of routine care or industry. Written informed consent for participation was not required from the participants or the participants’ legal guardians/next of kin in accordance with the national legislation and institutional requirements.

## Author contributions

LN: Conceptualization; Data curation; Formal analysis; Writing: original draft; review & editing. MCu: Formal analysis; Writing: original draft; review & editing. GC: review & editing. CS: Data curation; Formal analysis, review & editing. EC: Conceptualization; review & editing. AM-M: Data curation; Formal analysis, review & editing. AH: review & editing. MD: review & editing. TM: review & editing. MS-C: review & editing. MCo: review & editing. J-LM: review & editing. AE: Data curation; Formal analysis; review & editing. MS: Conceptualization; Data curation; Formal analysis; Writing: original draft; review & editing. All authors contributed to the article and approved the submitted version.
